# A Divalent Ion Is Crucial in the Structure and Dominant-Negative Function of ID Proteins, a Class of Helix-Loop-Helix Transcription Regulators

**DOI:** 10.1371/journal.pone.0048591

**Published:** 2012-10-30

**Authors:** Marie Vivian Wong, Sizun Jiang, Paaventhan Palasingam, Prasanna R. Kolatkar

**Affiliations:** 1 Laboratory for Structural Biochemistry, Genome Institute of Singapore, Singapore, Singapore; 2 Department of Biological Sciences, National University of Singapore, Singapore, Singapore; Wake Forest University, United States of America

## Abstract

Inhibitors of DNA binding and differentiation (ID) proteins, a dominant-negative group of helix-loop-helix (HLH) transcription regulators, are well-characterized key players in cellular fate determination during development in mammals as well as *Drosophila*. Although not oncogenes themselves, their upregulation by various oncogenic proteins (such as Ras, Myc) and their inhibitory effects on cell cycle proteins (such as pRb) hint at their possible roles in tumorigenesis. Furthermore, their potency as inhibitors of cellular differentiation, through their heterodimerization with subsequent inactivation of the ubiquitous E proteins, suggest possible novel roles in engineering induced pluripotent stem cells (iPSCs). We present the high-resolution 2.1Å crystal structure of ID2 (HLH domain), coupled with novel biochemical insights in the presence of a divalent ion, possibly calcium (Ca2+), in the loop of ID proteins, which appear to be crucial for the structure and activity of ID proteins. These new insights will pave the way for new rational drug designs, in addition to current synthetic peptide options, against this potent player in tumorigenesis as well as more efficient ways for stem cells reprogramming.

## Introduction

The ID proteins belong to the family of HLH transcription factors, however, their lack of a basic region accounts for their inability to bind to DNA, distinguishing them from the other basic HLH (bHLH) transcription factors [1]–[5]. The first ID gene, ID1, was initially discovered and isolated in 1990 by Benezra et al [1]. Various ID1 paralogs were subsequently discovered by other groups [2]–[5], and named ID2, ID3 and ID4. The crucial role of the ID proteins lies in their dominant-negative effect when forming heterodimers with other DNA-binding members of the HLH family, disrupting the protein-DNA interaction [1]. The mRNA expression of ID proteins is generally high during growth and development, but greatly reduced upon maturation of the cell [6]. It was hypothesized and subsequently validated that the varying levels of ID proteins, as well as other partner HLH transcription factors, were critical in determining the cell's eventual fate: directing them into a one-way, specialization or preventing them from differentiating [7]–[9]. Today, although the full complement of binding partners of the ID proteins have yet to be established, studies have shown that they are mainly bHLH transcription factors that fall into Class I of the HLH family [10]. Class I bHLH transcription factors, such as the E12 and E47, are generally ubiquitously expressed; Class II bHLH transcription factors, such as the myogenic and neurogenic proteins MyoD1 and NEUROD1, are usually tissue-specific [10]–[12]. Both classes of bHLH proteins have the ability to homo or heterodimerize on DNA containing the E-box consensus motif (CANNTG), such as that found in the muscle creatine kinase (MCK) enhancer [11], [13]. The balance between homodimerization and heterodimerization appears to have varying determinants on cell fate, triggering a variety of cellular differentiation pathways or posing as essential checkpoints in cellular fate regulation [8], [10]–[12]. By regulating the levels of Class I bHLH proteins, ID proteins can effectively control cellular fate by shifting the equilibrium for Class I:Class II bHLH heterodimer formation.

While ID genes are not considered oncogenes [14], their critical role in cancer is not to be overlooked; recent studies suggest strong association of ID proteins with tumor angiogenesis [14], [15]. Undoubtedly, ID proteins present an attractive target in novel anti-cancer treatments [14], [16]. The roles of ID proteins in the repression of embryonic and induced pluripotent stem cell differentiation are also well documented [9], [17], [18], highlighting the importance of ID proteins in the maintenance of stem cells. More interestingly, the overexpression of ID1 and ID2 appears to trigger cellular division and proliferation in terminally differentiated cells [19]. This dedifferentiation potential of ID proteins opens up an entirely new window of possibilities for iPSC programming, as seen in an interesting 2-step method utilizing only ID3 and Oct4 in mice [20].

**Figure 1:**
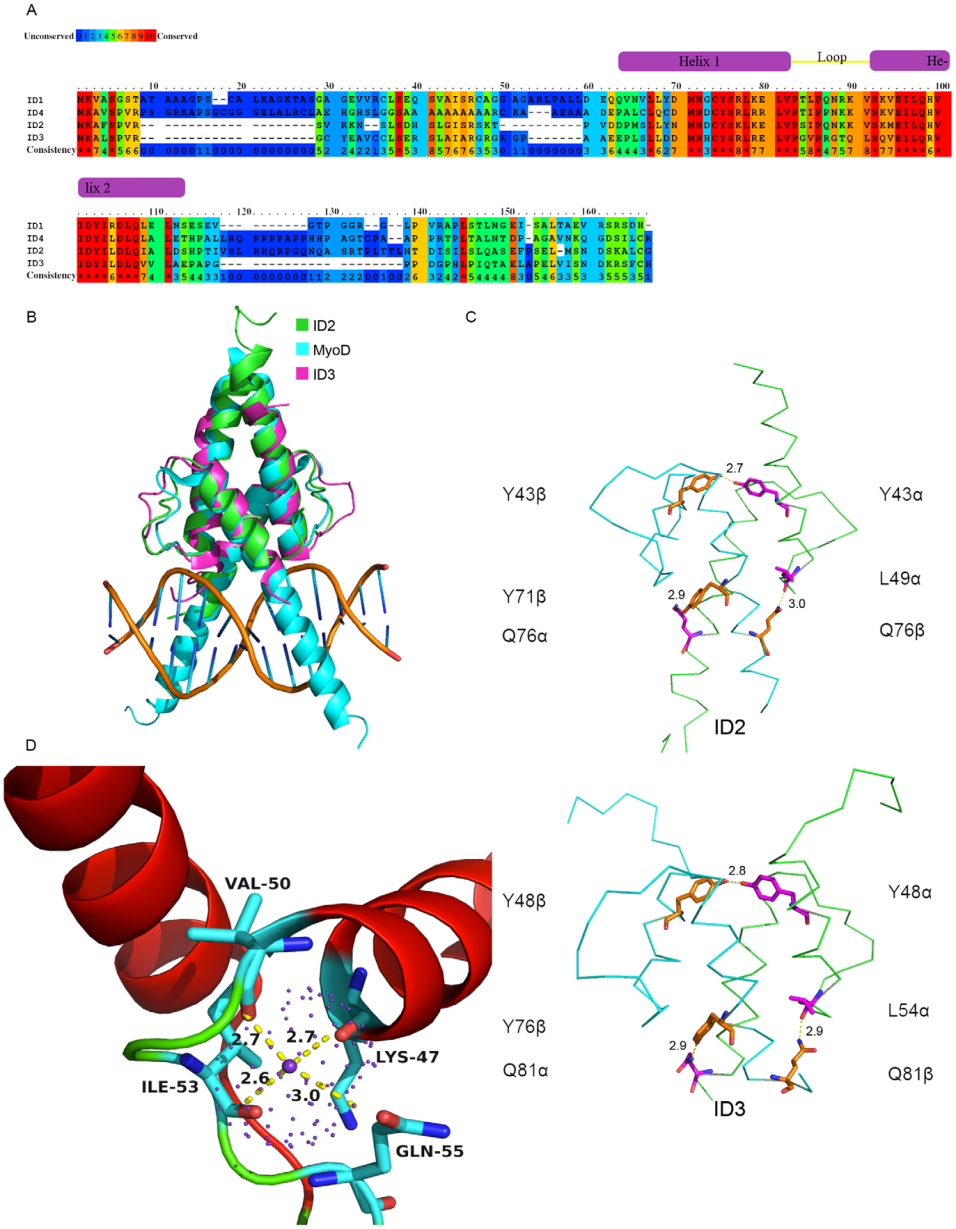
Structural Features of ID2 (A) Full length protein sequence alignments of ID1, ID2, ID3 and ID4 (GenBank accession numbers CAI20171.1 [ID1], NP_002157.2 [ID2], CAA51827.1 [ID3] and AAA73923.1 [ID4]). Alignments were performed using PRALINE [47], with the BLOSUM62 weights matrix and the ID proteins grouped according to sequence similarity. (B) Structural alignments of ID2, ID3 (PDB ID: 2LFH) and MyoD (PDB ID: 1MDY) helix-loop-helix regions. (C) Conserved hydrogen bonds in the ID2 and ID3 structures, involving Y43α-Y43β, L49α-Q76β and Q76α-Y71β for ID2, as well as their ID3 counterparts Y48α-Y48β, L54α-Q81β and Q81α-Y76β. (D) Observed positive ion in the loop of ID2, as well as the interacting amino acids K47, V50, I53 and Q55. Structural figures were generated usi [48] ngPyMol [49].

We present the novel homodimeric crystal structure of the HLH domain of the human ID2 determined at 2.1Å resolution. Our study further validates that ID proteins, as with their other HLH counterparts, appears to exist as homodimers in their active, native states, rather than monomers.

**Figure 2:**
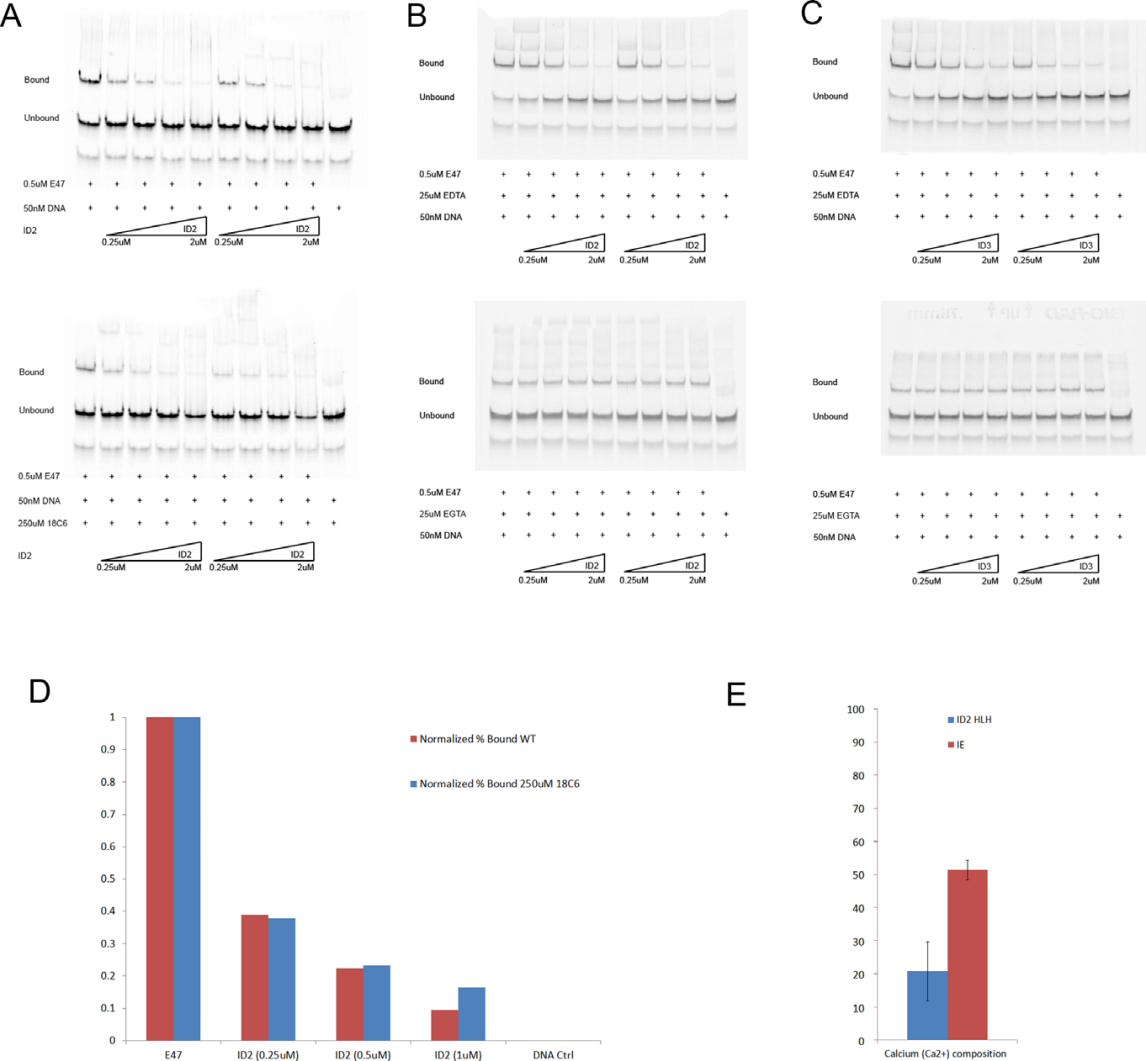
Identification of the positive ion in electrophoretic mobility shift (EMSA) assays. (A) Top: Wild type ID2 titrated against 0.5 uM E47. Bottom: Wild type ID2 titrated against 0.5 uM E47 in the presence of 250 uM 18C6. (B) Top: Wild type ID2 titrated against 0.5 uM E47 in the presence of 25 uM EDTA. Bottom: Wild type ID2 titrated against 0.5 uM E47 in the presence of 25 uM EGTA. (C) A repeat of (B) with ID3 instead of ID2. (D) Normalized quantification (to E47 Control for each lane; see Methods for details) of the ID2-E47 EMSAs in (A). (E) The y-axis represents calcium levels (uM) in ID2 and ID2-E47 complexes (IE), tested using the AbcamColormetric Calcium Detection Kit (See Methods), with 400 uM protein sample concentrations.

The loop of the ID proteins has been shown to be absolutely critical for their function [21]; we present the first known data indicating the presence of a divalent ion in the loop that interacts with 4 amino acids in ID2, and show that the knockdown of this ion will obliterate ID2 and ID3 activity. Hence, we propose the ion's essential role in maintaining the rigidity of the loop in ID proteins, and henceforth their structure and function.

**Figure 3:**
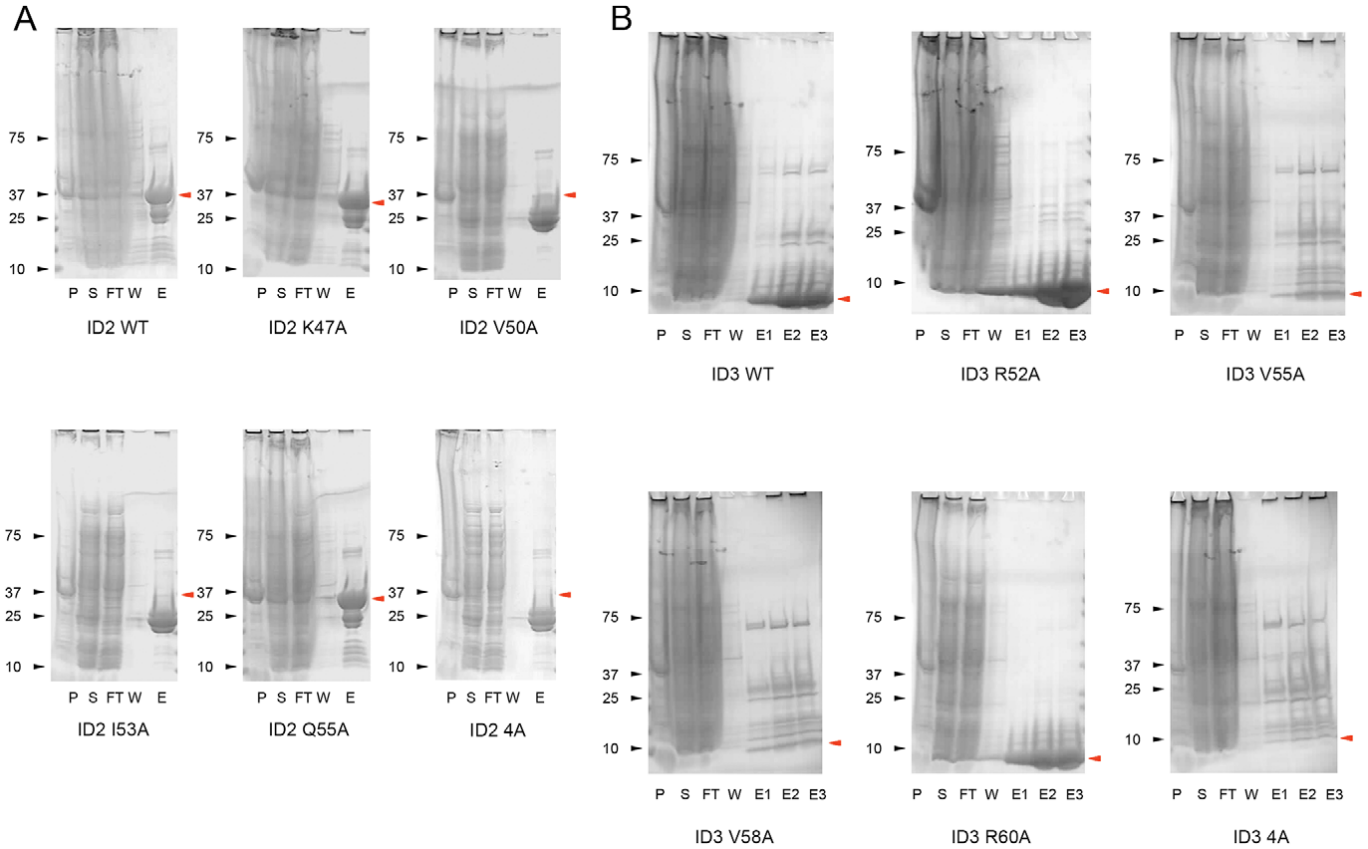
Protein expression profiles of ID2 and ID3 loop mutants in *E.coli*. ID2 (A) and ID3 (B) ion loop interacting amino acids were systematically mutated to alanine and the expression levels observed by SDS PAGE. 4A  =  all 4 amino acids simultaneously mutated to alanine. The rough molecular weight of the ID2-HisGST polypeptide is 37 kDa, while the ID2-His polypeptide is under 10 kDa, and their relevant sizes are indicated by the red arrows. Figure legend: P  =  Pellet, S  =  Supernatant, FT  =  Flowthrough from affinity column, E1, E2, E3  =  Eluates from the affinity column.

## Materials and Methods

All oligonucleotides were synthesized by 1^st^ Base Singapore and all PCR amplification was done with Takara *Ex Taq*, Clontech, unless otherwise specified. A comprehensive list of all primers used in the cloning and generation of mutants for ID2, ID3 and E47 are listed in Table S1.

**Figure 4:**
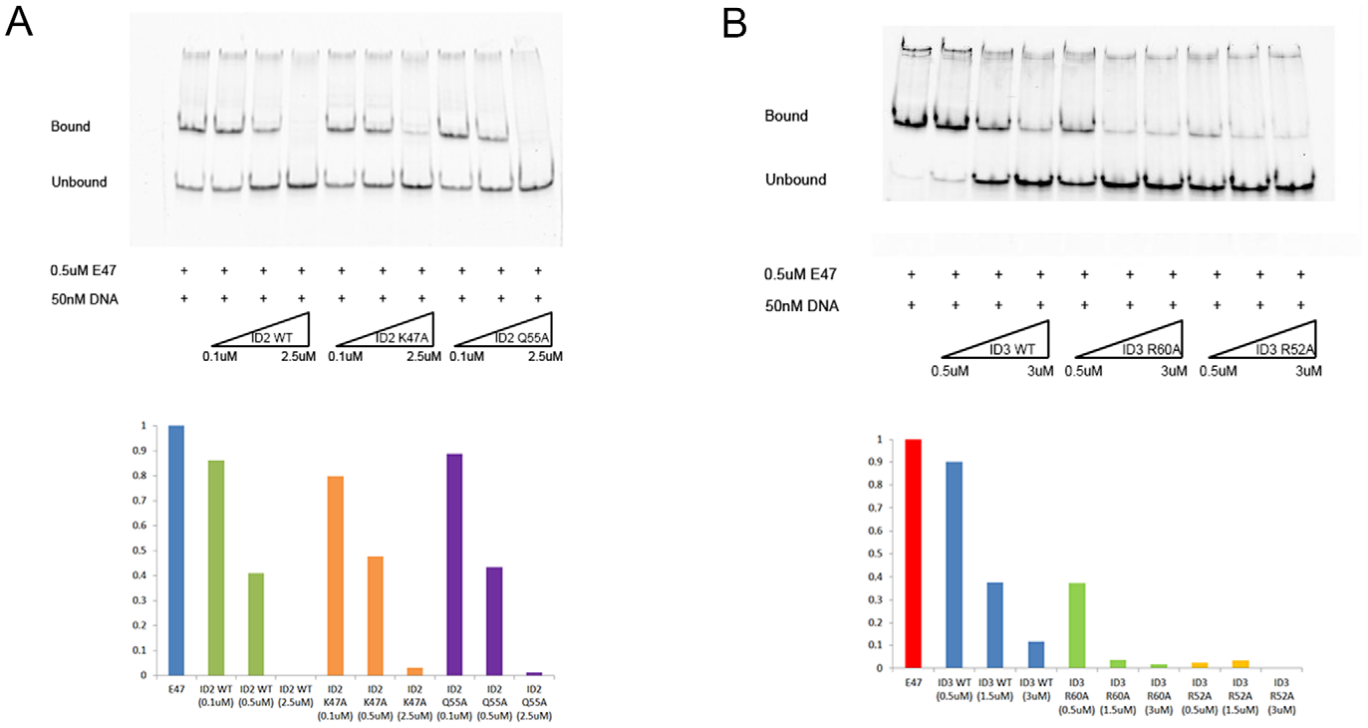
EMSAs of ID2 and ID3 loop mutants that could be expressed and purified. (A) Top: EMSA gel for titration of ID2 wild type (WT) and mutants (K47A, Q55A) against 0.5 uM of E47. Bottom: Normalized quantification of the EMSA gel on top, 2 replications. (B) A repeat of (A) with ID3 instead of ID2.

### Cloning of ID2, ID3, E47 and MyoD1 constructs for expression and crystallization

ID2 HLH (residues 24–82) was cloned from full-length cDNA (Genecopoeia, Z0585) using Gateway® (Invitrogen) as per manufacturer's instructions, into the pDONR221 vector (Invitrogen). Due to known instability issues of ID2, a C-terminal 14 amino acid long polypeptide (LKPSFLVQSGDIAS) was included to increase stability [22], and a Tobacco Etch Virus (TEV) protease cleavage site was included at the N terminus of ID2 HLH.

**Figure 5:**
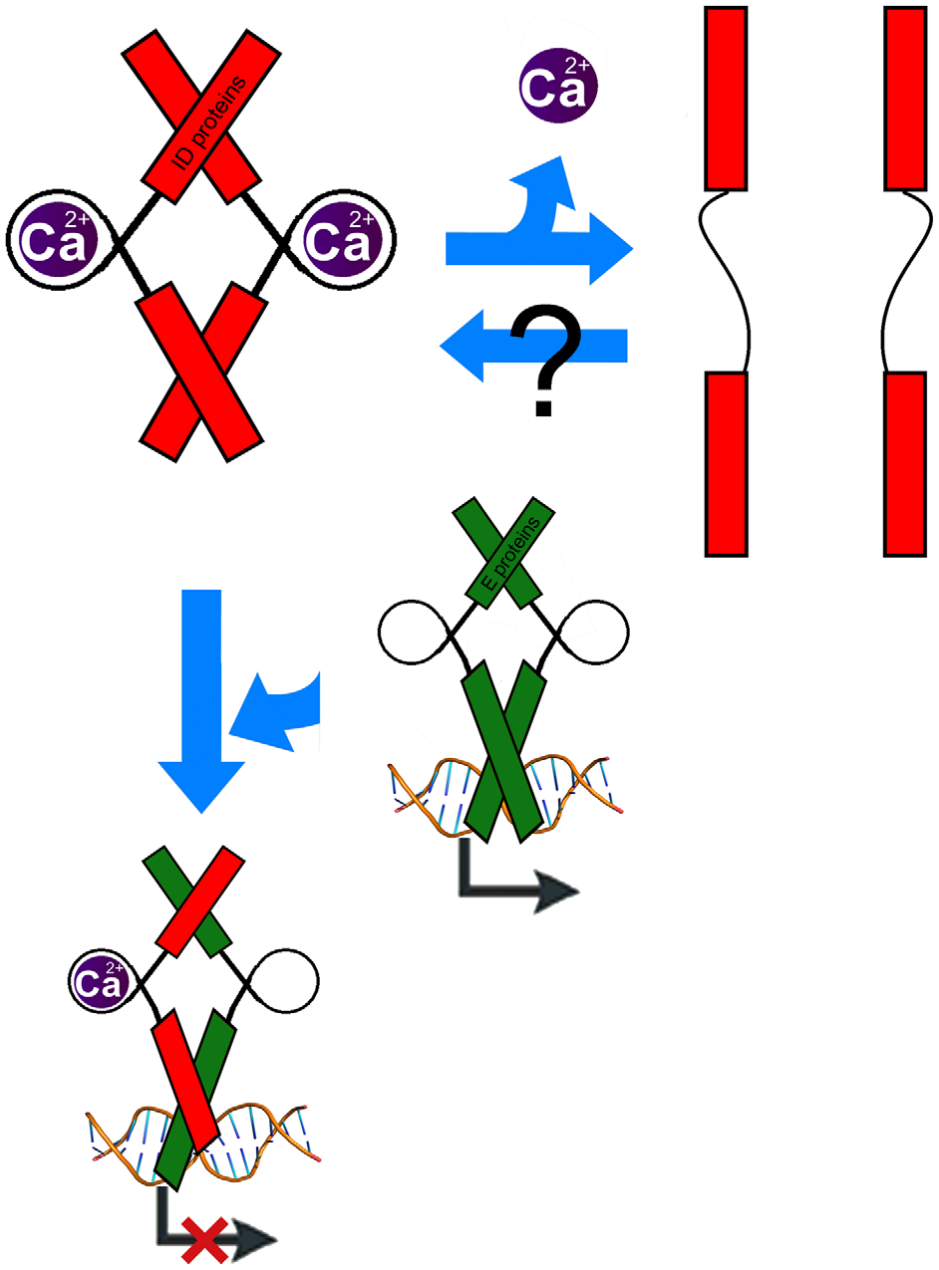
Proposed role of the Ca2+ ion in the structure and function of ID proteins. Ca2+ appears to have a role in allowing heterodimer recognition by the helical region of ID proteins. Addition of EGTA appears to inhibit heterdimerization, and hence the ability of ID proteins to recognize their binding partners.

ID3 HLH (residues 36–86), as defined by the ID3 NMR structure in the Protein Data Base (PDB ID 2LFH), was cloned from full-length cDNA (Genecopoeia, Z5773) into the pCOLADuet-1 vector (Novagen), with an N terminal hexahistidine tag (MGSSHHHHHHSQDP) present in the vector via a BamHI and NotI co-digestion (FastDigest, Fermantas) of the insert and vector.

E47 HLH (residues 545–606) was cloned from synthesized oligonucleotides (1^st^ Base Singapore) using Gateway® (Invitrogen) as per manufacturer's instructions into the pDONR221 vector (Invitrogen), and a Tobacco Etch Virus (TEV) protease cleavage site included on the N terminus of E47 HLH.

Once cloned inserts into pDONR221 vectors were verified by sequencing (1^st^ Base Singapore), they were cloned into expression vectors using Gateway® (Invitrogen) as per manufacturer's instructions. The final optimized expression vector for ID2 purification and subsequent crystallization was pDest-565 (Addgeneplasmid 11520), which contains an N-terminal His-GST tag. The final optimized expression vector for E47 HLH and MyoD1 HLH was pDest-HisMBP (Addgene plasmid 11085) [23].

All PCR and digestion results were confirmed on a 1% agarose gel (BioRad) run at 125V for 30 minutes before PCR purification (PCR Purification Kit, Qiagen).

All clones were transformed into *E. coli* TOP10 competent cells (Invitrogen) and screened with the relevant antibiotics before plasmid extraction via miniprep (Miniprep Kit, Qiagen) and Sanger sequencing (1^st^ Base Singapore), unless otherwise specified.

### Cloning of ID2 & E47 coexpression vector into pCOLADuet-1

Primers were designed (Table S1) for the PCR amplification of ID2 HLH to have a 3′ Bam HI and 5′ Not I cut site, while E47 HLH had a 3′ Nde I and 5′ Kpn I cut site. The pCOLADuet-1 vector included a start codon with an N terminal His-Tag and 4 amino acid linker (MGSSHHHHHHSQDP-) for ID2, while a C terminal Strep-Tag and 2 amino acid linker was included in the primer for E47 HLH (-SAWSHPQFEK Stop). ID2 HLH and pCOLADuet-1 were double digested with Bam HI and Not I (FastDigest, Fermentas) as per manufacturer's protocol, before ligation (Quick Ligation Kit, NEB) and subsequent transformation into E. coli TOP10 competent cells (Invitrogen). Successful clones were screened by Sanger sequencing (1st Base Singapore) before a double digestion with NdeI and Kpn I (FastDigest, Fermentas) ligation with the E47 HLH insert. Transformation and ligation and subsequent sequencing were performed as described above to confirm the successful cloning of both ID2 HLH and E47 HLH into pCOLADuet-1.

### Cloning of ID323

Primers (Table S1) were designed to amplify the helix 1 and 2 of ID3 separately via PCR, and PCR purified (PCR Purification Kit, Qiagen) individually. ID323, ID3 helixes with a ID2 loop replacement, were then generated using a bridge primer in addition to the forward and reverse primers, and cloned into pCOLADuet-1.

### Protein expression in *E.coli*

The expression plasmids was transformed into *Escherichia coli*BL21(DE3) competent cells (Stratagene). A single colony was picked and added to Luria Broth (LB) containing 100 µg/mL Ampicillin (pDest-565) or 50 µg/mL Kanamycin (pCOLADuet-1) and grown at 37°C overnight at 220 rpm in a temperature controlled shaker incubator (Innova 44R, New Brunswick). 20 ml of this overnight culture was then added to 580 ml of fresh media per 2L shaker flask (BellCo Glass) the next day, and grown at 37°C, 220 rpm. The cultures were induced with 0.5 mM IPTG after their OD reaches 0.6–0.8, and then allowed to grow overnight at 18°C, 220 rpm.

### Se-Met protein production for ID2 pDest 565 construct

A single colony was picked and grown in 5 mL LB with 100 µg/mL Ampicillin at 37°C overnight the shaker incubator (Innova 44R, New Brunswick) at 220 rpm. The culture was centrifuged at 2000 rpm for 5 mins, the pellet resuspended in 5 mL M9 minimal media (12.8 g/L Na_2_HPO_4_-7H_2_O, 3.1 g/L KH_2_PO_4,_ 0.5 g/L NaCl, 0.5 g/L MgSO_4,_ 0.1 mM CaCl_2,_ 5g/L NH_4_Cl, 20% d-Glucose) and the process repeated once before resuspending again in 2 mL M9 media. 150 mL of M9 media was then added to the culture and allowed to grow overnight at 37°C, before addition to fresh M9 media as described above. At OD 0.6, an amino acid mix (100 mg K, F, and T; 50 mg I, L, and V; and 60 mg SeMet) was added to each liter of culture and mixed for 10 mins at 37°C. The resulting culture was then induced with 0.4 mM IPTG at 18°C overnight. More detailed methods are described in [22].

### Cell Harvesting

Cells were harvested by ultracentrifugation at 10,000 rpm for 10 mins at 4°C with the SLA-3000 rotor in a Sorval 5C centrifuge (ThermoScientific). The pellets were resuspended in lysis buffer (50 mM Tris, 300 mM NaCl, 30 mM Imidazole, pH 7.3 (ID2 constructs) or pH 8 (ID3 constructs, E47 HLH and MyoD1 HLH)) and subjected to ultrasonication (Sonic Dismembrator Model 500, Fischer Scientific) for 7 mins at 35% amplitude, 2 seconds pulses, on ice. The sample was then centrifuged at 19,000 rpm for 1 hour at 4°C, with the SS-34 rotor (ThermoScientific) to remove any cell debris, before filtration through a 0.2 µm polyethersulfone (PES) membrane (Nalgene Fast PES Filter, ThermoScientific), and the supernatant collected. All buffers contained 5 mM DTT for the Se-Met proteins.

### Protein purification and identification

Manual protein purification was performed with NiNTA beads (Qiagen) as per manufacturer's protocol, with 1 ml of final bead volume used per 7.2L of culture for pCOLADuet-1, and 2 ml of final bead volume per 1.6L of culture for pDest-565. Beads were equilibrated in lysis buffer before crude lysate was passed through columns (Econo-Pac® Chromatography columns, BioRad) for His-Tag pulldown. Elution buffer (50 mM Tris, 300 mM NaCl, 300 mM Imidazole, pH8.0 for ID3 and ID323 constructs, IE and E47 HLH; pH 7.3 for ID2 and constructs) was used to elute proteins bound to the NiNTA beads.

In the case of ID2His-GST and E47-MBP, the protein eluate was mixed with 1∶100 (by concentration) of TEV protease at 4°C overnight, ensuring that Imidazole concentrations were below 60 mM via buffer dilution with the desalting buffer (50 mM Tris, 100 mM NaCl). A final ion exchange purification step was performed on the ÄKTA Express system (GE Healthcare) with a Resource S 6 ml column (GE Healthcare) and an increasing salt gradient (A1 buffer: 50 mM Tris, 100 mM NaCl; B1 buffer: 50 mM Tris, 1 M NaCl; pH 7.3 [ID2] or pH 8.0 [E47]).

In the case of ID3 and ID323, the NiNTA bead eluates were done in 1 ml aliquots, before analysis via SDS-PAGE for ID3 expression. The aliquots containing high amounts of target protein (as determined by MW) were pooled to 5 ml before loading into the ÄKTA Express system (GE Healthcare) for gel filtration using a Superdex 200 column (GE Healthcare) (Equilibration buffer: 50 mM Tris, 300 mM NaCl, pH 8.0).

In the case of IE (ID2-E47 complex), NiNTA bead eluates were subsequently loaded onto StrepTactin beads (StrepTactin Sepharose High Performance, GE Healthcare) as per manufacturer's protocol. Eluates were then collected in 1 ml aliquots.

The eluted protein fractions were verified by SDS-PAGE before being pooled, buffer exchanged (50 mM Tris pH 8.0, 300 mM NaCl) and concentrated using a membrane-based concentrator with a 3000 Da MW cutoff (Vivaspin, Sartorius), as per manufacturer's protocol. Protein concentrations were quantified using nanodrop (NanoDrop 1000, ThermoScientific) with parameters as determined by ProtParam [24]. 50 µL aliquots of each protein of 90% purity or higher were stored at −80°C at a concentration of 1 mg/ml or higher. We sometimes have trouble with reproducibility when quantifying ID2 HLH constructs on the nanodrop, hence the MicroBCA protein assay kit (Pierce Biotechnology) was used for ID2 HLH.

Purified proteins were analyzed via SDS-PAGE were subject to an in gel digestion and extraction (In-gel Tryptic Digestion Kit, ThermoScientific), before mass spectroscopy analysis (LTQ, ThermoScientific). The resulting output was searched using Mascot Daemon.

### Crystallization, data collection and structure determination

Crystals were obtained by hanging drop vapor diffusion. 1 uL of protein solution (7 mg/ml) in 50 mM Tris pH 8.0, 100 mM NaCl was mixed with 1 uL precipitant solution (0.1 M MES pH 6.5, 2.0 M Potassium Acetate for ID2-Nterm-HLH and 3 M Ammonium Acetate for ID2-HLH-Se-Met (crystals looked similar to those of ID2-Nterm-HLH). Crystals were flash frozen in liquid nitrogen prior to data collection. A 3.0Å resolution SAD dataset at Peak (12,658.3 eV) wavelength for ID2-HLH-semet was collected at the Argonne National Laboratory synchrotron, GM/CA-CAT, Sector 23, beam line ID-D equipped with a MAR300 CCD detector. ID2-Nterm-HLH native dataset at 2.1Å resolution was collected at the Brookhaven National Laboratory synchrotron on the X29 beamline equipped with an ADSC Q315r detector. The SAD dataset was indexed, and integrated in MOSFLM [25] and scaled in SCALA (CCP4 suite) [26]. The native dataset was processed using HKL2000 [27].

### Structure solution and refinement

Although a MAD dataset was collected at 3 wavelengths: Peak (12,658.3 eV), Inflection (12,656.5 eV), and Remote (13,058.3 eV), the structure of ID2-HLH-semet was solved using only the peak wavelength. Four selenium sites of ID2-HLH-semet were Identified using SOLVE in PHENIX [28] at a resolution range of 50–2.5Å. The structure was refined using PHENIX.REFINE to a 3.0Å resolution and used as a starting model for molecular replacement using AUTOMR (PHENIX) of the ID2-Nterm-HLH native dataset.AUTOBUILD (PHENIX) was performed on the AUTOMR coordinates and the rest of the model was manually built into 2Fo–Fc and Fo-Fc maps using COOT [29]. Model bias was monitored using simulated annealing composite omit map calculated at the start of the refinement using CNS [30]. Ten percentof the reflections were randomly assigned to the Rfree set for cross-validation. Further refinement was done manually by iterating through X,Y,Z coordinates and isotropic B-factor cycles using PHENIX.REFINE. The final model was composed of a 4-helix bundle resolved at 2.1Å. PyMol [31] was used for generating the figures in this paper. A summary of the data collection and refinement statistics can be found in Table S2.

### Calcium Detection

Calcium detection in purified ID2 and ID2-E47 (IE) complexes were performed as using the Colorimetric Calcium Detection Kit (ab102505, Abcam), as described in the manufacturer's protocol with small modifications. The protein equilibration buffer (50 mM Tris, 300 mM NaCl, pH 8.0) was used instead of ddH2O to dilute the calcium standards, and also used as a blank to verify that absence of a baseline of Ca2+ in the buffer. ID2 and IE protein samples of 400 uM concentrations were used. Protein samples were denatured for 5 minutes at 95°C before a 1 minute spindown at 16,100 g (Eppendorf 5415 R). In short, kit reagents were mixed as per protocol with protein samples before incubating in the dark for 10 minutes. Their OD_575_ was subsequently measured on a Spectramax M5 microplate reader (Molecular Devices). Readings were done in triplicates.

### Site-directed mutagenesis

Site-directed mutagenesis (QuikChange II XL Kit, Stratagene) was performed as per manufacturer's instructions. Specific primers were constructed using the QuikChange Primer Design software provided in the kit (Table S1).

### Electrophoretic mobility shift assay

Electrophoretic mobility shift assays (EMSA) were performed as described previously [32], [33] with some modifications. 5′-Cy5-labelled E-box DNA probe 5′-GGATCCCCCCAA**CACCTG**CTGCCTGA-3′ and mutant e-box probe 5′-GGATCCCCCCAA**ACTGGT**CTGCCTGA-3′ (Sigma, Proligo) with their exact reverse complements were annealed in a thermocycler (BioRad). Proteins were serially diluted and incubated for 10 mins at room temperature in the protein native buffer (50 mM Tris, 300 mM NaCl, pH 8.0). Cy5-labelled probe and binding buffer (20 mM Tris pH 8, 50 mM KCl, 1 mM DTT, 1 mM EDTA, 10% glycerol, 0.1 mg/mL BSA) added for an additional 15 mins at room temperature to make up a total of 20 ul per sample. 10 ul of each sample was loaded onto a 10 well 6% Tris-glycine native polyacrylamIde gel and subject to electrophoresis in a 1xTris-Glycine buffer (25 mMTris pH 8.3, 192 mM Glycine) at 4°C for 30 minutes at 200V, before visualization using a Typhoon 9140 PhosphorImager (Amersham Biosciences).

The free and bound DNA were quantified using ImageQuant TL software (Amersham Biosciences, GE Healthcare), and the ratio of bound DNA was tabulated for each individual lane with the following equation:
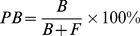
PB  =  Percentage bound (%), B  =  Bound DNA, F  =  Free DNA.

Normalization of PB DNA was done with the following equation:
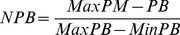
NPB  =  Normalized Percentage bound, MaxPB  =  Max Percentage bound, MinPB  =  Min Percentage bound.

### EGTA, EDTA and 18C6 knockdowns of ID2 and ID3 in EMSAs

Stock solutions of the chemicals were prepared (0.5 M for EGTA [Sigma-Aldrich] and EDTA [1^st^ Base], 5 M for 18C6 [Sigma-Aldrich]). EMSAs were performed as described above, with the addition of 1 ul of stock solution to the incubation mix of ID and E proteins. The incubation time (10 min, 15 min) were adhered to, as was the final volume of the sample (20 ul).

## Results

### Overall structure of ID2 as a homodimer

We have solved the structure of the ID2 homodimer HLH domain (residues 24–82) at a 2.1Å resolution, coupled to a stabilizing 14 amino acid linker region (LKPSFLVQSGDIAS). The asymmetrical ID2 structure contains one homodimer consisting of α & β monomeric chains different predominantly in the loop regions.

The topology of ID2 is, as expected, similar to other bHLH proteins such as E47, MyoD and NeuroD [34]–[36], with the same parallel, four-helix bundles (Figure 1b). The main difference is the absence of the extended helical basic region in ID2 compared to bHLHs, hence ID2 is highly unlikely to bind to DNA. We report the unprecedented presence of a positively charged ion that appears to hold the loop in place by interacting with 4 amino acids in or near the loop (Figure 1d).

The final model of the ID2 homodimer contains a 54 residue α-chain and a 47 residue β-chain representing the HLH domain. The boundaries of the HLH domain in ID2 are residues 33–82 on the α-chain and residues 40–82 on the β-chain.

### ID2 and ID3 HLH Dimeric Interface comprising of M39, L47, L49, M62, I69, I72, L75 and M44, L51, L54, V67, I74, I77, L80 respective

The dimeric interface is held together by a hydrophobic core consisting of residues M39, L46, L49, M62, I69, I72, L75 (Figure S1); all of which have equivalent reported buried residues in the structures of E47 [36], MyoD [35], E47-NeuroD [34] and ID3 [37]. The corresponding ID3 dimeric interface residues, M44, L51, L54, V67, I74, I77, L80, align closely with their counterparts in ID2, making up the hydrophobic core of the ID3 dimerization interface.

Besides the hydrophobic core, there are also three inter-chain hydrogen bonds at Y43α-Y43β, L49α-Q76β and Q76α-Y71β (Figure 1c). An equivalent contact for Q76α-Y71β is found in the E47-NeuroD structure at E47.E596-NeuroD.Y149, while equivalent contacts for all three hydrogen bonds are found in ID3 (Figure 1c). To assess the importance of this conserved hydrogen bond in ID2 heterodimer formation, site directed mutagenesis was performed on Y71 and Q76, resulting in the single mutations Y71A, Y71D, Q76A and Q76F. Any of these mutations resulted in complete loss of soluble protein expression (Results not shown). It is highly likely that these interdimeric hydrogen bonds are critical in holding the ID2 homodimer together, in addition to the hydrophobic core, providing additional stability to the ID2 homodimer.

The ID3/ID1 homology models [38] showed potential ID2 homodimer interactions as follows: N38 repulsing K61, D41 hydrogen bonding with Q71 and K45 forming a salt bridge with D75. However, these predictions were not observed in our structure, nor in the ID3 structure [37]. Interestingly, they show the predicted interaction of ID3.Y76-Q81 (which corresponds to ID2.Y71-Q76) in their models but it is not highlighted in the text. Both these interactions are observed in ID2 and ID3 structures (Figure 1c).

In another earlier work, a cysteine residue in helix-1 of ID2 was shown to be critical in homodimer formation by the creation of a hypothesized intermolecular disulfide bond [39]. However, our structure reveals C42 on each monomer pointing away from each other (Figure S2); their distant proximity does not allow the formation of a disulfide bond. This is also confirmed in the ID3 structure. However, we do not discount the fact that this cysteine may play a role in bringing the monomers closer together to form the functional homodimer via transitional interactions during homodimerization.

### The presence and role of a divalent ion in the loop region

One of the most striking observations in our ID2 structure is the presence of a positively charged ion near the N terminus of the loop, the first observation of its kind. At 2.5σ, the radius of the electron density is approximately 1.4Å. The size and the interactions of the ion with the side chain oxygen molecules of K47, V50, I53 and Q55 (with distances less than 3.5Å) suggest that this ion most likely corresponds to that of potassium (K+). The K+ ion, as well as its interacting amino acids, is mirrored in both monomers of ID2 (Figure 1d). We postulate that the presence of a positive ion the loop of ID proteins could account for its rigidity, as indicated in previous studies [21], where the mutagenesis of 2 residues in ID1 (L76, and Q78) led to loss in MyoD binding affinity. Not surprising, L76 and Q78 in ID1 corresponds to I53 and Q55 in ID2 respectively, both involved in interactions with the K+ ion. We observe that the main chain oxygen of K47, V50 and I53 were responsible for the interaction with the K+ ion, whereas the interaction of Q55 with the ion was via the side chain oxygen.

To investigate the possible role of K+ in ID2 structure and function, 18-Crown-6 (18C6) (Sigma-Aldrich) was used to sequester the K+ cation with high specificity and affinity [40]. Purified ID2 was incubated for 10 minutes with E47 in the presence of 25 uM of 18C6 per lane, and subsequently loaded on a 6% TG gel as described in Methods. There were no noticeable effects of 18C6 on ID activity (Figures 2a and 2d).

The buffer that ID2 crystallized in contained 2 M of Potassium Acetate, hence we considered the possibility of a displacement of the native ion with K+. Calcium/calmodulin inhibition of bHLH transcription factors (reviewed in [41]) suggested alternate ions to test. 25 uM of ethylene glycol tetraacetic acid (EGTA) and ethylenediaminetetraacetic acid (EDTA) were each incubated with ID2 and ID3, and their activities observed via EMSA (Figure 2b and 2c). EGTA appeared to have an irreversible and detrimental effect on the activity of both ID3 and ID3, while EDTA did not have a noticeable effect. This suggests the presence of a divalent ion, likely calcium, being involved in the activity of ID proteins.

Due to the high similarity in both the structure and sequence of the HLH domain of ID2 and ID3, we repeated the experiment with ID3, with similar results (Figure 2b and 2c). We hypothesize conserved ionic interactions in both the loops of ID2 and ID3 (ID2: K47, V50, I53, Q55; ID3: R52, V55, V58, R60).

### Calcium levels in ID2 and IE

Calcium levels in ID2 and the coexpressed ID2+E47 complex (IE) were quantified as described in Methods. Readings were performed in triplicates, and protein equilibrium buffers were used as blanks as well as verified for the absence of a baseline Ca2+ level. Results of the assay are shown in Figure 2e. As expected, IE calcium levels were significantly higher than ID2 calcium levels due to the sequential pulldown of ID2 and E47 (see Methods), which selected for highly active protein fractions of ID2. Addition of 5 mM of CaCl2 to *E.coli BL21 (DE3)* also appeared to result in higher yields of recombinant ID protein (S.J. and P.R.K., unpublished results).

### ID2 and ID3 mutant studies on the 4 interaction amino acids with the ion

Alanine scanning was performed on the 4 amino acids involved in the loop for both ID2 and ID3, and expressed as described in Methods. The expression profile of all the WT proteins and their respective mutants are shown (Figure 3a and 3b). The poor expression levels of ID2.V50A, ID2.I53A and ID3.V55A, ID3.V58A resulted in unrecoverable recombinant protein when expressed in *E.coli*. However, ID2.K47A, ID2.Q55A and ID3.R52A, ID3.R60A had WT like expression levels, and could be further purified for EMSA studies. The expressible mutants also had similar or even higher activity compared to WT ID2 and ID3 *in vitro* (Fig. 4a and 4b). Expectedly, when all 4 amino acids were mutated simultaneously, no protein expression was seen (Fig. 3a and 3b).

The loop of ID2 was swapped into ID3 (hereby termed ID323) to try to determine the loop of ID2 was essential for its specificity and activity ID3. We did not detect substantial binding differences between ID3 and ID323 to E47 (Figure S3).

## Discussion

The HLH domain of ID is nearly sufficient for its dimerization and activity [21], as with MyoD and E47 [42], [43]. Interestingly, in the initial studies of ID proteins, the authors all inevitably concluded or accepted that although ID proteins had high affinities for heterodimerization, they homodimerized poorly, unlike the rest of the HLH family [2], [21], [44]. Based on the structural homologies in the HLH family, Wibley et al. attempted to create a 3D homology model of ID3 [38], postulating that ID proteins could homodimerize even without DNA for stability due to hydrophobic core packing. A cysteine residue was also determined as absolutely crucial for the dimerization and function of ID2 [39], leading to the postulation of a disulphide bond involved in the ID homodimerization. We have showed that ID2 appears to exist as homodimers in its native state, and the absence of a disulphide bond in the final structure of ID2.

Our novel report of a divalent ion, possibly Ca2+, influencing the inhibitory interactions of ID proteins present a striking similarity to calmodulin-mediated bHLH inhibitions, discovered in 1994 by Grundstrom and colleagues [45]. ID-calmodulin interactions, direct or indirect, have yet to be reported. Calmodulin and S-100 proteins seem to bind to bHLH proteins at their DNA binding basic regions, which ID proteins lack. Future work in this area will be needed, as there seems a redundancy of Ca2+ dependent ID and calmodulin inhibition of bHLH targets, although the targeted proteins may be slightly different.ID proteins are upregulated by many known oncogenes (such as Ras, Myc and ETC); unsurprisingly, overexpression of ID proteins are also seen in many tumors [14]. Indeed, ID proteins are now seen as attractive drug targets for therapy of some cancers. Perhaps sequestering calcium levels in these tumor cells would result in lower activity of ID proteins, hence lower rates of stimulation of self-renewal and tumorigenesis.

The current structural and biochemical studies of ID2 and ID3 reinforce previous studies on the critical role of the loop in the dominant-negative activity of ID proteins. Recent studies of the ID-like protein, Human homologue of murine maternal ID-like molecule (HHM), a dominant-negative inhibitor of the Class II bHLH Olig1, shows the potential role of the helical N and C terminal bundles in the autoinhibition and possible stabilization of HHM, which appears to be a monomer in solution due to a lack of contact of the helixes of its HLH domain [46]. Due to the absence of N and C terminal bundles in ID proteins, the presence of an ion in the loop of the HLH domain may serve as a scaffold to hold the loop rigidly in place by pulling the helix 1 and helix 2 of ID proteins closer to allow for hydrophobic intramolecular and intermolecular interactions to occur, therefore accounting for the stability and native homodimer configuration of ID proteins (Figure 5). In the cases of the non-expressible mutants (ID2:V50A & I53A; ID3: V55A & V58A), the unrecoverable protein indicates that these mutants were probably misfolded, and we offer the explanation that one possible contributor would be the absence of a scaffolding ion.

We do not exclude the possibility that EGTA is affecting direct amino-acid interactions between the loop of ID and unmapped regions of E47. However, this model does not explain the phenomena seen in the EGTA/EDTA inhibition assays. Additionally, in current structures of ID, the loop regions do not appear to be involved in direct binding of their heterodimeric partners. Novel structures of ID heterodimers would add more evidence to this possible role of the ID loop. Further work could comprise validation of the model and additional experiments to understand how the actual ions are modulated *in vivo*, and whether the process is reversible.

### Accession Number

Coordinates for the ID2 HLH homodimer have been submitted to the Protein Database (PDB) with the accession number 4AYA.

## Supporting Information

Figure S1 The amino acids (blue and yellow) involved in the hydrophobic homodimeric core of ID2.
(TIF)

Figure S2 The locations of the C42s in the homodimeric ID2. The structure strongly suggests that a C42–C42 disulphide bond is highly unlikely to be formed in the final homodimeric form of ID2, although such an interaction maybe be possible in a transient state.
(TIF)

Figure S3 An EMSA gel and its quantification showing the interactions of the ID3 HLH domain (lanes 4–6) and the ID323 fusion protein (ID3 helix 1, ID2 loop, ID3 helix2; lanes 1–3) against E47. The experiment was performed as described in Methods.
(TIF)

Table S1 A list of the primers used in this study.
(XLSX)

Table S2 The crystallographic data collection and refinement statistics of the ID2 homodimer.
(XLSX)
