# Supplementary figures and images for: A Divalent Ion Is Crucial in the Structure and Dominant-Negative Function of ID Proteins, a Class of Helix-Loop-Helix Transcription Regulators
Source: PLoS One. 2012 Oct 30;7(10):e48591. doi: 10.1371/journal.pone.0048591 (PMC3484135; doi:10.1371/journal.pone.0048591)

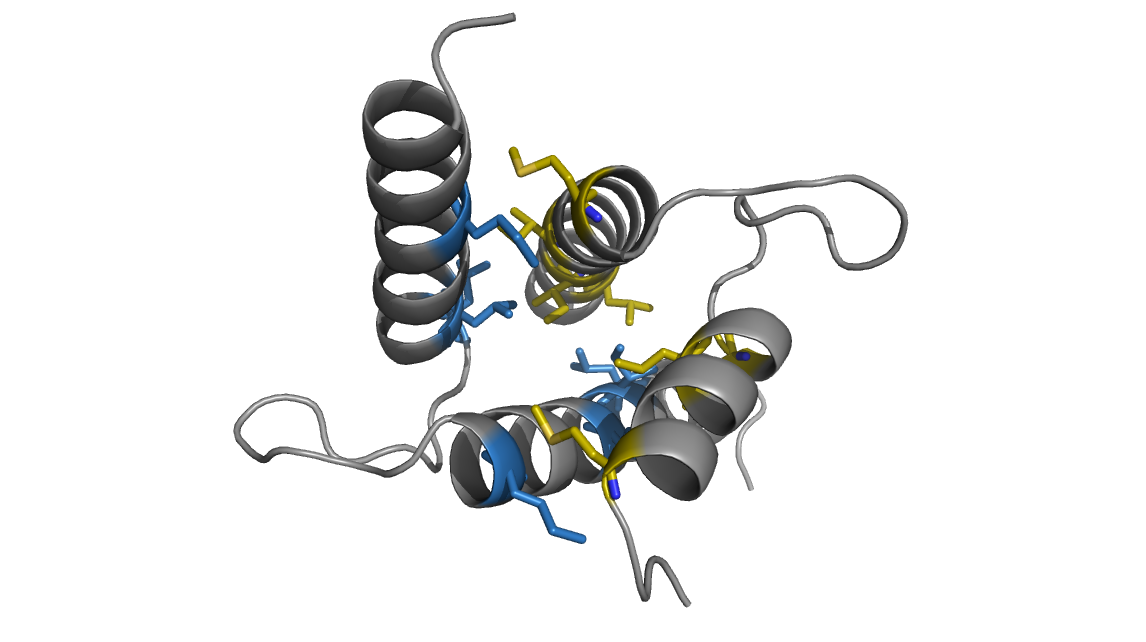

Supplement: Figure S1 — The amino acids (blue and yellow) involved in the hydrophobic homodimeric core of ID2. (TIF) [file pone.0048591.s001.tif]

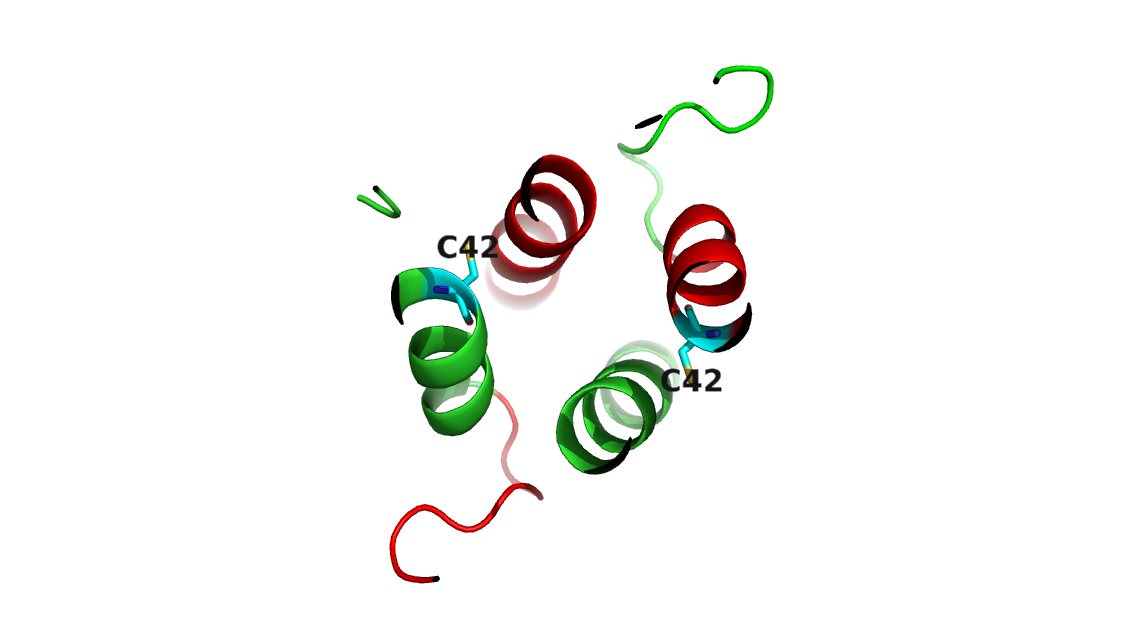

Supplement: Figure S2 — The locations of the C42s in the homodimeric ID2. The structure strongly suggests that a C42–C42 disulphide bond is highly unlikely to be formed in the final homodimeric form of ID2, although such an interaction maybe be possible in a transient state. (TIF) [file pone.0048591.s002.tif]

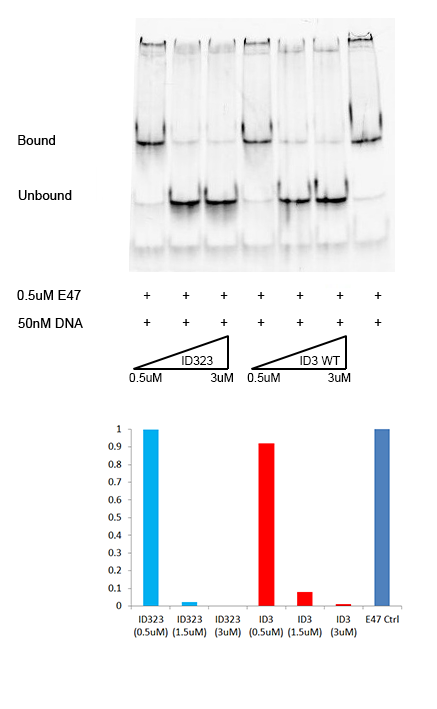

Supplement: Figure S3 — An EMSA gel and its quantification showing the interactions of the ID3 HLH domain (lanes 4–6) and the ID323 fusion protein (ID3 helix 1, ID2 loop, ID3 helix2; lanes 1–3) against E47. The experiment was performed as described in Methods. (TIF) [file pone.0048591.s003.tif]
